# Low‐frequency mechanical vibration induces apoptosis of A431 epidermoid carcinoma cells

**DOI:** 10.1002/elsc.201900154

**Published:** 2020-02-27

**Authors:** Wresti L. Anggayasti, Chikahiro Imashiro, Taiki Kuribara, Kiichiro Totani, Kenjiro Takemura

**Affiliations:** ^1^ Department of Chemical Engineering, Faculty of Engineering Brawijaya University Malang Indonesia; ^2^ Department of Mechanical Engineering Keio University Yokohama Kanagawa Japan; ^3^ Department of Materials and Life Science, Faculty of Science and Technology Seikei University Tokyo Japan

**Keywords:** apoptosis, cancer cell model, glucose metabolism, high‐mobility group box 1, mechanical vibration

## Abstract

Cancer research is increasingly focused on discovering strategies to induce cancer cell apoptosis without affecting surrounding normal cells. One potential biocompatible method is mechanical vibration, which has been developed as part of the emerging field of mechanomedicine. Previous studies of mechanical vibration have employed high‐frequency vibration, which damages healthy cells. In this study, we examined the effects of brief (1 h) low‐frequency (20 Hz) mechanical vibration on glucose consumption and survival (apoptosis, necrosis, HMGB1 release) of the human epidermoid carcinoma cell line A431. We found that apoptosis, but not necrosis, was significantly increased at 48 h after mechanical vibration compared with cells maintained in static culture. In keeping with this, extracellular release of HMGB1, a necrosis marker, was lower in cultures of A431 cells subjected to mechanical vibration compared with control cells. Glucose consumption was increased in the first 24 h after mechanical vibration but returned to control levels before the onset of apoptosis. Although the precise intracellular mechanisms by which low‐frequency mechanical vibration triggers apoptosis of A431 cells is unknown, these results suggest a possible role for metabolic pathways. Mechanical vibration may thus represent a novel application of mechanomedicine to cancer therapy.

**Abbreviation**
HMGB1: high‐mobility group box 1

## 1 INTRODUCTION

Cells are capable of managing their own repair and renewal, and even defending themselves. These properties depend on their response to many kinds of stimuli from the surrounding microenvironment, including external mechanical forces 1. Cells translate external physical stimuli into biochemical and biophysical responses via signaling pathways involving mechanosensory proteins and the cytoskeletal network, a process known as mechanotransduction 2. A recent review by Naruse described a number of medical therapies based on mechanotransduction, including manipulation of ion channels sensitive to mechanical stimuli, manipulation of tissue constructs for the treatment of heart failure, and various uses in regenerative medicine. This review introduced the term “mechanomedicine” as a new field of therapy that uses mechanotransduction to improve health 3. Various studies have described the benefits of mechanomedicine to health, such as promoting bone formation in an osteoporotic mouse model 4, stimulating wound healing in mice with diabetes 5, controlling pressure in vivo to suppress cancer metastasis 6, and enhancing gene expression 7. In each of these studies, mechanical vibration was used as the stimulus in vivo.

Conventional cancer chemotherapies often induce harmful adverse effects 8, suggesting a need for novel biocompatible approaches to treatment. One potential therapy is mechanical vibration, which has been investigated in several studies. High‐frequency mechanical vibration can successfully kill cancer cells, but it also injures nearby healthy cells 9. Some studies have examined whole‐body vibration as an adjuvant to traditional chemotherapy and shown that it reduces several side effects of chemotherapy, such as weight loss and nausea 10. However, there is currently no evidence that whole‐body vibration alone has a direct impact on cancer cells.

During carcinogenesis, cell death via autophagy and apoptosis is limited and the predominant mechanism of death is necrosis 11. However, promotion of necrotic cell death as a means of cancer therapy is undesirable because it induces leakage of cell contents into the surrounding environment, which may trigger inflammatory responses that promote tumor progression. Therefore, induction of cancer cell death by apoptosis would be preferable to reduce the possibility of inflammation and immune cell activation 8, 12. Mechanical vibration of anisotropic magnetic nanoparticles attached to cell membranes has been shown to trigger apoptosis at subkilohertz frequencies 8. Although the study by Leulmi et al. 8 was performed in vitro, it successfully demonstrated that cell fate can be controlled by mechanical vibration at frequencies as low as 20 Hz, which is important because an increase in temperature is unlikely to be elicited at such a low frequency. Moreover, the risk of damage or induction of necrosis in surrounding healthy cells by heat diffusion is also reduced 13. However, because that study involved magnetic particles and application of a magnetic field, the contribution of mechanical vibration to the observed increase in apoptosis was unclear 8.

To address some of the questions surrounding mechanical vibration as a potential cancer therapy, in this study we examined the effect of mechanical vibration on cancer cell metabolism and death using the human epidermoid cancer cell line A431 as a model. We compared the responses of cells subjected to a short period of mechanical vibration at 20 Hz with control cells incubated without mechanical stimulation. In addition to apoptosis and necrosis, we examined glucose consumption, which is closely related to nutrient deprivation in cancer, increasing at early stages and slowing down when cells undergo apoptosis 14, and high‐mobility group box 1 (HMGB1) protein release, which is a feature of necrosis 15. We found that mechanical vibration induced an increase in glucose consumption and apoptosis, but not in either HMGB1 release or necrosis, compared with control conditions. Thus, mechanical vibration may represent a potential strategy to induce apoptosis of cancer cells while minimizing cell death by necrosis.

**PRACTICAL APPLICATION**

Previous research highlighted the use of high‐frequency vibration could trigger cell cancer death. However, this method also damages the surrounding healthy cells. This study has observed that the application of low‐frequency mechanical vibration induces apoptotic cell death and not necrosis, upregulates glucose consumption, and lowers the release of necrosis marker high mobility group box 1 (HMGB1). The overall results suggest the involvement of metabolic pathways. This study potentially represents novel application of mechanomedicine in cancer therapy.

## 2 MATERIALS AND METHODS

### 2.1 A431 cell culture

The human epidermoid carcinoma cell line A431, which is considered a good model to study the biomechanical response of cancer cells to extracellular stimuli 16, was obtained from RIKEN (Wako, Saitama, Japan). Cells were cultured in DMEM containing 3.7 g/L sodium bicarbonate, 4.5 g/L glucose, 4 mM glutamine (1196509; Gibco, Tokyo, Japan), and 10% (v/v) fetal bovine serum for 24 h at 37°C in a 5% CO_2_ atmosphere. Before experiments, cells were incubated for 48 h in the same medium lacking fetal bovine serum.

### 2.2 Mechanical stimulation and measurement of output vibration

**Figure 1:**
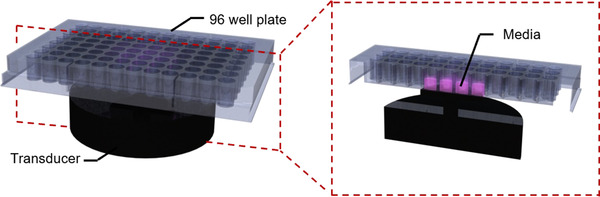
Experimental setup for cell vibration. Mechanical vibration was applied to cells in a 96‐well plate placed on the vibration transducer and secured with double‐sided tape. Cells were seeded in wells in the center of the plate. Left and right images show side and cross‐sectional views, respectively

A speaker‐based vibration system was used to mechanically stimulate A431 cells. The system consisted of a vibration transducer (VLN‐S8BT; Veilnet, Osaka, Japan) connected via Bluetooth to the controller software (Pd‐extended) on a personal computer. Mechanical stimulation was applied in sinusoidal waves to the bottom of the 96‐well plate, which was fixed to the vibration transducer as shown in Figure 1.

The excitation vibration frequency was set to 20 Hz, and the vibration amplitude in each well was evaluated and calibrated using a laser Doppler vibrometer (LV‐1800; Onosokki, Yokohama, Japan).

### 2.3 Cell vibration

**Figure 2:**
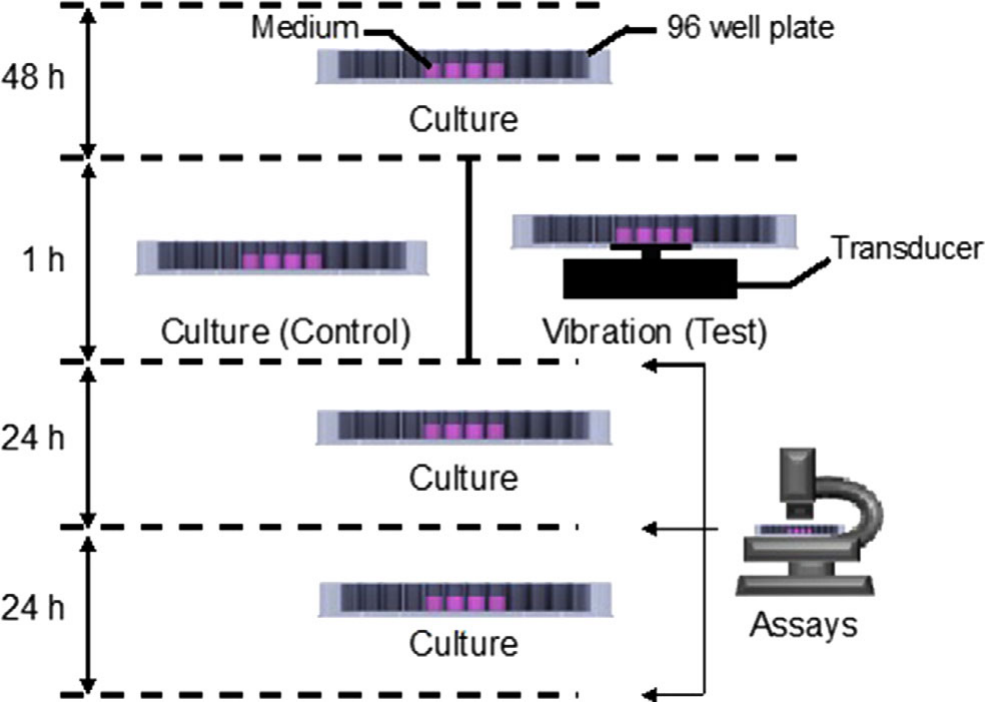
Schematic illustration of experimental procedures. Cells were cultured for 48 h and then either incubated for an additional 1 h (control cells) or exposed to mechanical vibration for 1 h (test plates). The cells were then collected and subjected to assays immediately (0 h) or after 24‐ or 48‐h incubation in static culture

**Figure 3:**
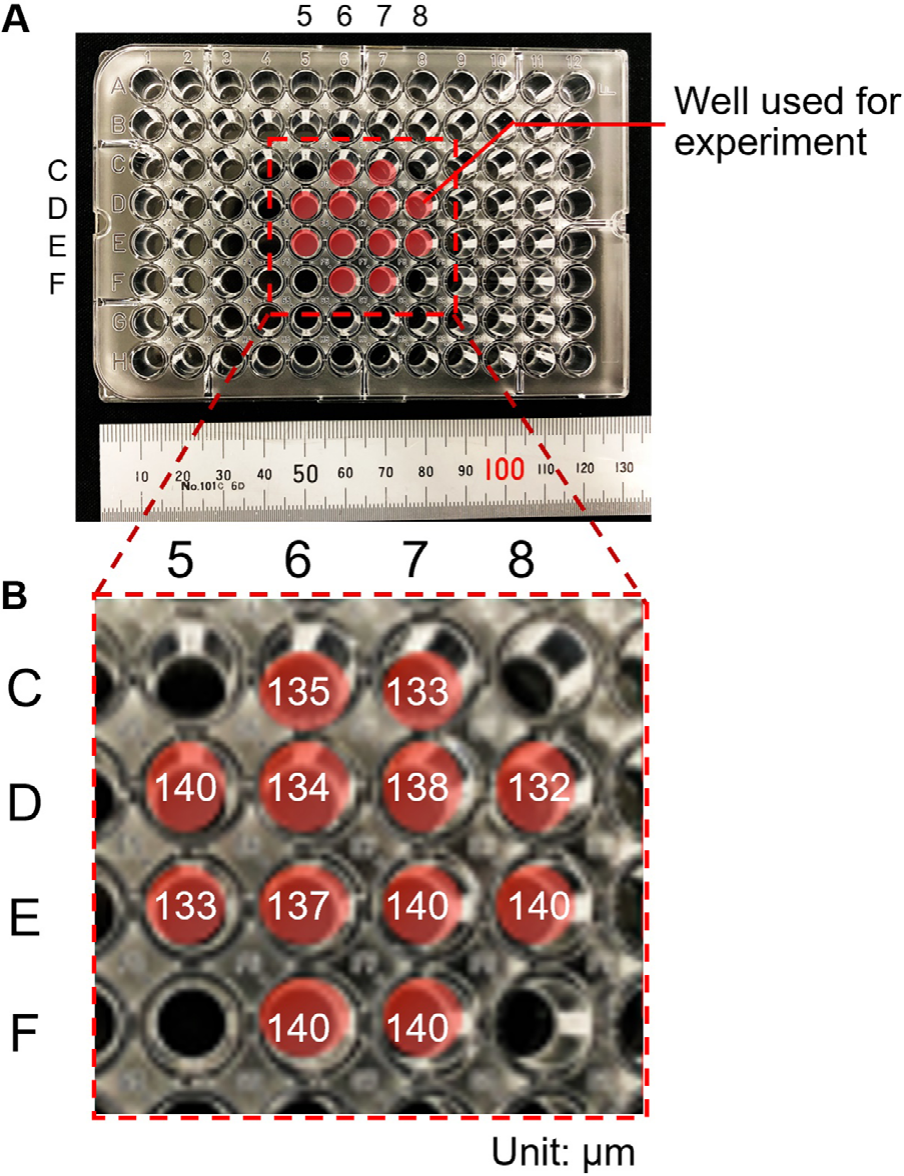
Distribution of vibration displacement in experimental wells. (A) Plate image showing the wells used for the experiments. (B) Enlarged view of the central wells showing the vibration amplitude (µm) of each well. The maximum vibration amplitude was 140 µm

Cells were seeded into 12 wells at the center of the 96‐well plate (Figure 3) at a density of 1.5 × 10^5^/250 µL/well in serum‐free DMEM and cultured for 48 h. The entire experimental system was then placed in a humidified CO_2_ incubator for 1 h at 34°C. The medium temperature was confirmed to reach 37°C during the vibration period due to the heat generated by the vibration transducer. After the 1‐h vibration, test plates were removed from the system and cultured without vibration for 1, 24, or 48 h as described below (Figure 2). Control plates were incubated without mechanical vibration for the same times.

### 2.4 Apoptosis and necrosis assay

A431 cell death was analyzed at 0, 24, and 48 h after mechanical vibration using an Apoptosis/Necrosis Detection Kit (ab176749; Abcam, Cambridge, UK) according to the manufacturer's instructions. This kit enables discrimination between apoptotic and necrotic cells. The cells were visualized with an Eclipse Ti‐S inverted microscope (Nikon, Tokyo, Japan) and images were captured using NIS‐Elements D version 4.30 software followed by processing and quantification of live/dead cells with ImageJ software (National Institutes of Health, Bethesda, MD, USA).

### 2.5 Glucose consumption assay

Cell supernatants were collected from the test and control wells at the indicated times after mechanical vibration. Glucose concentrations were determined using a Glucose Assay Kit (GAHK‐20; Sigma‐Aldrich, St. Louis, MO, USA) according to the manufacturer's instructions.

### 2.6 HMGB1 ELISA

HMGB1 release was measured using an HMGB1 ELISA kit (6010; Chondrex, Redmond, WA, USA) according to the manufacturer's instructions. In brief, cell supernatants were collected at the indicated times and pooled. ELISA plates were coated with capture antibody for 21 h at 4°C and then washed. Aliquots (50 µL/well) of diluted standards or supernatant samples were added to the wells, mixed with the detection antibody solution, and then incubated for 1 h at 37°C followed by 19 h at 4°C. The wells were washed, incubated with streptavidin‐peroxidase solution for 30 min at 25°C, and washed again. For detection of peroxidase activity, 3,3′,5,5′‐tetramethylbenzidine was added to the wells for 30 min at 25°C, and 2 N sulfuric acid was then added to terminate the reaction. OD_450_ values were measured using a FlexStation3 microplate reader (Molecular Devices, San Jose, CA, USA). Released HMGB1 was quantified by comparison with a standard curve constructed with recombinant HMGB1 protein (0.8–50 ng/mL).

### 2.7 Statistical analysis

Data are presented as the mean ± SD of biological triplicates. Differences between group means were evaluated by Student's *t*‐test. *P* < 0.05 was considered statistically significant.

## 3 RESULTS

### 3.1 Characterization of mechanical vibration

To assess the effects of mechanical vibration on A431 cell metabolism and survival, cells were seeded into flat‐bottomed 96‐well plates and exposed to vibration at a frequency of 20 Hz for 1 h. The vibration amplitude was shown to be approximately homogeneous in wells in the center of the plate, with a maximum amplitude of 140 µm (Figure 3). The vibration amplitude was measured at the center of each well, which is reasonable because the possible vibration wavelength in a well subjected to 20 Hz vibration is longer than 100 m. Based on these findings, experiments were conducted on cells cultured in the 12 central wells (Figure 3).

### 3.2 Vibration at 20 Hz promotes A431 cell apoptosis

**Figure 4:**
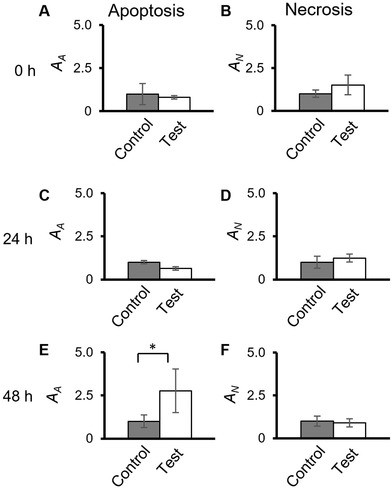
Mechanical vibration induces apoptosis but not necrosis of A431 cells. (A, C, and E) Area of apoptotic A431 cells (A_A_) normalized to the value for control cells immediately after mechanical vibration (0 h, A) or after 24‐h (B) and 48‐h (C) incubation. (B, D, and F) As described for (A), (C), and (E), except the area of necrotic cells normalized to the control cells (A_N_) is shown. Data are presented as the mean ± SD of *n* = 3. *P < 0.05 by Student's *t*‐test

The impact of mechanical vibration at 20 Hz for 1 h on A431 cell death was analyzed by assessing both apoptosis and necrosis. The proportion of control and mechanically stimulated cells undergoing apoptosis or necrosis was not significantly different at 0 h (immediately after vibration) or after 24 h incubation (Figure 4A,C). However, apoptosis was significantly higher for mechanically stimulated cells than control cells at 48 h (Figure 4E). In contrast to apoptosis, the proportion of test and control cells undergoing necrosis was not significantly different at any time point (Figure 4B, D, and F).

### 3.3 Glucose consumption by A431 cells is transiently increased by mechanical vibration

**Figure 5:**
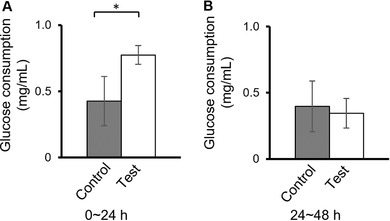
Mechanical vibration increases glucose consumption by A431 cells. Glucose uptake in culture supernatants was measured between 0 h and 24 h (A) or between 24 h and 48 h (B) after mechanical vibration. Data are presented as the mean ± SD of *n* = 3

Glucose consumption by A431 cells was assessed by measuring the glucose concentration in the culture supernatant between 0 and 24 h and then between 24 and 48 h after treatment. As shown in Figure 5, cells subjected to mechanical vibration showed significantly increased glucose consumption compared with control cells during the first 24 h posttreatment (Figure 5A), whereas consumption was not significantly different between 24‐ and 48‐h incubation (Figure 5B).

### 3.4 Mechanical vibration does not increase HMGB1 release by A431 cells

**Figure 6:**
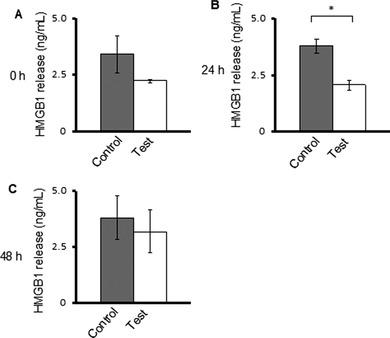
Mechanical vibration does not increase HMGB1 release from A431 cells. HMGB1 levels in culture supernatants were analyzed by ELISA immediately after 0 h (A), or 24 h (B) and 48 h (C) after vibration. Data are presented as the mean ± SD of *n* = 3

HMGB1 is known to be actively secreted by apoptotic cells and passively released by necrotic cells, and its release thus serves as a marker of cell stress and death. We found that HMGB1 levels were not significantly different in the culture supernatants of control or mechanically stimulated A431 cells at 0 and 48 h incubation but were significantly higher in control cells at 24 h (Figure 6).

## 4 DISCUSSION

The results of this study reveal that mechanical vibration of A431 cancer cells increases glucose consumption over the first 24 h following vibration and results in death by apoptosis, but not necrosis, within 48 h. HMGB1 release was not increased by mechanical vibration; indeed, mechanically stimulated cells released lower levels of HMGB1 than did control cells. Various studies have shown that cells can withstand some degree of mechanical vibration 17, 18 but excessive heat generation can cause necrosis. In this study, because a normal culture temperature was maintained during vibration treatment, we can assume the cell death resulted from mechanotransduction.

The mechanism by which mechanical vibration promotes cancer cell death had previously been unclear. Leulmi *et al*. showed that magneto‐mechanical vibration induces apoptosis of renal cancer cells 8, although the net effect of mechanical vibration in triggering apoptosis in that study was unknown. Here, we demonstrated that pure mechanical vibration, without addition of magnetic particles or exposure to a magnetic field, can also induce apoptosis of cancer cells.

One mechanism by which cancer cells support their elevated rates of growth and proliferation is by fermentation of glucose; a phenomenon known as the Warburg effect 14. We found that mechanical stimulation increased glucose consumption by A431 cells in the following 24 h. Our results are consistent with many studies reporting that mechanical vibration induces cell to ingest increasing amount of nutrients, particularly glucose albeit through unknown mechanisms. This increasing glucose uptake is shown to be especially useful for cell differentiation, proliferation, migration, and extracellular matrix generation 19, 20, 21, 22. Our results are also consistent with previous studies demonstrating that depletion of glucose may lead to apoptosis of cancer cells 14, 23, although the direct link between the glucose consumption and upregulated cellular metabolism still needs to be scrutinized further.

In this regard, we found that mechanical vibration stimulated glucose uptake only in the first 24 h and decreased to control levels between 24 and 48 h. This reduction in glucose consumption later after mechanical vibration may be related to the significant rise in apoptosis observed at 48 h. Cancer cells exposed to metabolic stress (e.g., glucose deprivation) either activate cell survival signaling cascades or undergo cell death 11. Previous work has suggested that glucose consumption decreases after initiation of signaling for apoptosis 14, which may explain the relationship between the kinetics of glucose metabolism and apoptosis in our study.

We selected HMGB1 as a second marker of cell death in A431 cells subjected to mechanical vibration. HMGB1 is mainly released by necrotic cells and, to a lesser extent, by apoptotic cells 15, and the presence of extracellular HMGB1 thus signals cellular stress, necrosis, and tissue damage 24. Kang et al. proposed that HMGB1 secreted into the extracellular environment contributes to changes in tumor cell metabolism 23. HMGB1 interacts with a number of proteins and subsequently triggers intracellular signaling cascades in response to changes in the physicochemical environment 15, 25. For example, HMGB1 associates with the receptor of advanced glycation end products (RAGE) in cancer 15. This interaction is related to several bioenergetic pathways, particularly the PI3K/PTEN/AKT, LKB1/AMPK, and STAT3 pathways 23. A431 cells were previously shown to express and secrete HMGB1 26, 27.

Interestingly, we found that less HMGB1 was released from cells subjected to mechanical vibration compared with the control cells at all time points, and the difference was statistically significant at 24 h. The half‐life of HMGB1 in culture supernatants of cancer cells has been measured at 3 h 28. It corresponds with our result and discussion of HMGB1 release right after the stimulation (0 h). Since the half‐life of HMGB1 released by cancer cells is 3 h, the differences of the amount of HMGB1 released between the test and control samples after 1 h of mechanical vibration treatment is not negligible.

In conclusion, our results demonstrate that mechanical vibration at 20 Hz promotes the apoptosis of A431 cancer cells, which may be related to changes in the rate of glucose metabolism and/or HMGB1 release by the cells. The intracellular mechanisms underlying the effects of mechanical vibration on apoptosis are still largely unknown. However, our results suggest that a number of disparate pathways may converge to promote cancer cell death, and this merits further investigation. Future studies should also examine these effects in other cell and tissue types, whether cancerous or healthy ones, and in animal models. Previous work has established that mechanical vibration can halt the progression of several inflammation‐related diseases, such as diabetes and osteoporosis, in animal models 4, 5. Thus, mechanical vibration may have utility for the treatment of cancer, representing a new application for biomechanical manipulation in medicine.

## CONFLICT OF INTEREST

The authors have declared no conflict of interest.
